# Attention Deficit Hyperactivity Disorder: What Are Pharmacists’ Roles and Associated Outcomes?

**DOI:** 10.3390/ijerph20032754

**Published:** 2023-02-03

**Authors:** Mohamed Hassan Elnaem, Merna Mahmoud AbouKhatwa, Mahmoud E. Elrggal, Inderpal Singh Dehele

**Affiliations:** 1 School of Pharmaceutical Sciences, Universiti Sains Malaysia, Minden 11800, Malaysia; 2 Department of Clinical Pharmacy and Pharmacy Practice, Faculty of Pharmacy, Alexandria University, Alexandria 5372066, Egypt; 3 College of Pharmacy, Umm Al-Qura University, Makkah 21955, Saudi Arabia; 4 School of Pharmacy, University of Birmingham, Birmingham B15 2TT, UK

**Keywords:** pharmacist, attention deficit hyperactivity disorder, role, pharmaceutical care, outcomes

## Abstract

Globally, the prevalence of attention deficit hyperactivity disorder (ADHD) is increasing. The treatment for ADHD is multifaceted and requires long-term care and support. Pharmacists are capable of assisting patients and their caretakers in achieving desired outcomes. This work discusses and summarizes pharmacists’ roles in ADHD care and their associated outcomes. Overall, pharmacists are positioned to educate on ADHD, optimize medications in a collaborative practice model, manage and monitor side effects, and provide remote and virtual pharmaceutical care. Pharmacists could directly contribute to ensuring medication safety and increasing awareness regarding the optimal use of ADHD medications. Patients with ADHD can benefit from pharmacist involvement in a variety of ways, including, but not limited to, initial screening and referral, the provision of clinical consultation and feedback, and the improvement of self-management and self-awareness of the illness. Pharmacists also play a significant role in therapeutic decision making regarding the initiation, intensification, and monitoring of ADHD treatment to ensure its effectiveness and quality of life improvement. Lastly, pharmacists could help identify more cost-effective treatment approaches for ADHD patients based on the clinical scenario that is encountered.

## 1. Introduction

Over the last few decades, there has been an exponential increase in the overall prevalence of mental disorders [1]. Globally, healthcare professionals use the Diagnostic and Statistical Manual of Mental Disorders, Fifth Edition (DSM-V), as an official guide to classify and diagnose mental disorders [2]. It provides a shared vocabulary for healthcare professionals to discuss with their patients and offers concise and precise criteria to objectively assess symptom presentations in various clinical settings [2].

Patients with attention deficit hyperactivity disorder (ADHD) may experience a variety of adverse outcomes, and they are more likely to have children with ADHD due to the genetic components of the condition [3]. There is also a genetic link that could explain the co-existence of other neuropsychiatric disorders among cases of ADHD [4]. Furthermore, several factors related to parents, such as maternal polycystic ovary syndrome, were found to increase the odds of ADHD in their offspring, according to a recent meta-analysis [5]. Moreover, the development of ADHD outcomes was affected by the parenting stress that modulates adverse childhood experiences, especially among families with low resilience levels [6].

ADHD has a chronic impact on patients’ social and functional abilities and their risk of developing other comorbidities [7]. Adults with ADHD have a higher rate of unemployment and turnover, and they are more likely to have tried multiple careers before finding one that suits them [3]. In addition, adults with persistent ADHD symptoms who have not been treated with medication are at a higher risk of drug and substance dependence.

Because of their more frequent interactions with patients, pharmacists seem to be among the first healthcare professionals to encounter ADHD patients in their practice, particularly in community settings [7]. Pharmacists are increasingly seeing patients with neuropsychiatric illnesses; when properly trained, they feel more confident intervening to improve the care delivered to these patients [8]. When patients of varying ages express concerns about their prescribed ADHD pharmacotherapies, pharmacists should reassure them regarding the role of pharmacotherapy in controlling their condition [7]. The contribution of pharmacists to the clinical care of ADHD patients has been associated with improvements in the quality of care [9]. Given the overall increase in ADHD prevalence and the valuable care pharmacists could provide to ADHD patients, the purpose of this paper is to discuss and highlight the most recent evidence supporting pharmacists’ roles in the clinical care of ADHD patients and the outcomes associated with these roles.

## 2. ADHD Treatment

The goal of ADHD treatment is to enhance the patient’s functioning, learning, and ADHD symptoms [10]. The treatment available for ADHD includes behavioral, dietary, and pharmacotherapy interventions. In this section, essential information regarding the different treatment approaches is presented.

### 2.1. Behavioral Approaches

Psychoeducation, a therapeutic ADHD-focused parent-training program that provides information, support, and coping skills to the patient and their family, is recommended as the first-line intervention, particularly in children under five years [11]. In a randomized control study by Daley et al. involving 57 children, parenting programs improved parenting efficacy and child social performance [12]. The dissemination of information about ADHD is vital for spreading awareness and understanding the disorder, thereby overcoming the stigma towards ADHD patients. Parents aware of ADHD are more likely to accept and adhere to their child’s treatment [13]. In addition, by incorporating elements of the theory of planned behaviors, psychoeducation was found to have a positive impact on parents’ knowledge about ADHD and improve their intention to adhere to prescribed pharmacotherapy [14]. For better clinical outcomes, evidence has shown that pharmacists can adopt and design psychoeducational programs to improve medication adherence among patients with mental disorders in the primary care setting [15].

Furthermore, cognitive behavioral therapy (CBT) is a behavioral therapy that recognizes and targets specific maladaptive cognitions and behaviors that cause psychological distress and dysfunction [16]. Psychoeducation, organizational/planning, distractibility, adaptive thinking, procrastination, and relapse prevention are all covered in CBT sessions, which should be led by qualified professionals [17]. A randomized controlled study involving 26 adults with ADHD measured the efficacy of CBT. The findings highlighted that both younger and older adults receiving CBT showed significant improvement regarding inattention, executive dysfunction, and comorbidity [18]. The success of CBT in lowering the severity of symptoms is probably because CBT teaches patients specific strategies to tackle these thinking patterns and promote positive behavioral changes.

Neurofeedback, often known as “NF”, involves measuring an individual’s brain waves and giving them a feedback signal to educate them on how to exercise self-control over their brain activities [19]. A randomized controlled trial found that NF significantly reduced ADHD symptoms, which remained consistent at a six-month follow-up [20]. However, there is insufficient evidence to support the use of NF in the standard treatment of ADHD.

### 2.2. Dietary Approaches

Dietary modification for ADHD is divided into elimination and supplementation diets. The elimination diet excludes artificial food coloring, additives, sugar, and artificial sweeteners, and the few-foods diet (FFD) relies on a small number of food items (two types of meat, two sources of carbohydrates, two vegetables, two fruits, oil, and water) for a definite period [21]. The evidence to support the long-term effectiveness of FFD is inconclusive [11].

On the other hand, a supplementation diet involves the addition of amino acids, essential fatty acids, vitamins, and minerals. In a study that included 47 newly ADHD-diagnosed children and investigated the impact of a 5-week dietary modification program, the findings showed that dietary modifications, including hypoallergenic foods, a wide range of vegetables, and reduced carbohydrate and protein intake, were associated with improvement in ADHD symptoms [22]. Despite these findings, there is a lack of evidence to support the recommendation of specific dietary modifications as part of a standard ADHD treatment plan.

Overall, with quality education and training related to dietary supplements, pharmacists could have a crucial role in supporting appropriate and safe dietary supplement use among diverse groups of patients, including those with neuropsychiatric conditions [23]. However, this particular role has to be supported by clear professional responsibilities and resources to guide pharmacists’ safe and practical involvement in dietary supplement use in the community [24]. Table 1 outlines the overall treatment approaches for ADHD.

### 2.3. ADHD Pharmacotherapy

ADHD pharmacotherapy includes several therapeutic choices, including stimulants such as methylphenidate and amphetamine, noradrenergic drugs such as atomoxetine, and alpha-2A-adrenergic agonists such as guanfacine and clonidine (Table 2). For children aged five years and older and young people with ADHD, methylphenidate is the first line of treatment [11]. In general, atomoxetine and methylphenidate have similar effectiveness in improving social adjustment in youths with ADHD [25]. In another study by Roh and Kim, it was found that patients tolerate the osmotic-controlled oral delivery system (OROS) of methylphenidate better than extended-release methylphenidate, immediate-release methylphenidate, or atomoxetine [26]. In addition, parents were also more satisfied with the OROS methylphenidate formulation. The satisfaction was rated using the Satisfaction with Medication Scale and detailed questions about symptom severity, dysfunction, and quality of life. However, for patients who cannot tolerate methylphenidate or do not respond after six weeks of treatment with methylphenidate, after alternative preparations and adequate doses have been considered, atomoxetine can be used [11]. Table 2 shows essential information concerning the pharmacotherapy of ADHD [27].

## 3. Pharmacists’ Roles

**Figure 1:**
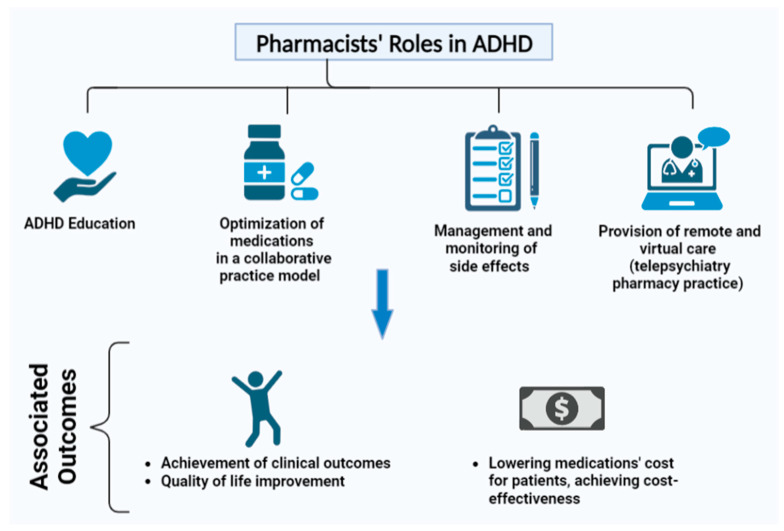
Pharmacists’ roles and associated outcomes in ADHD.

In general, clinical pharmacy gains more attention and characterizes pharmacists’ main role in providing services such as medication reconciliation, therapeutic education, and taking part in multidisciplinary teams to help optimize pharmacotherapeutic decisions for patients with neuropsychiatric disorders [28]. Concerning ADHD, pharmacists can play various roles in providing pharmaceutical care to patients with ADHD of various ages or at various stages of the disorder. Before confirming the diagnosis, pharmacists could notice potential ADHD symptoms through casual conversation or a more comprehensive consultation with a patient or caretaker and then refer the patient to receive a more formal evaluation from medical specialists. Following an ADHD diagnosis, the pharmacist can be a valuable resource for the patient regarding information and support, particularly if the patient or family struggles with a new diagnosis of ADHD due to the stigma linked to mental disorders or the stigma associated with using ADHD medication [7]. Pharmacists are also in the right place to provide medication management services for patients with ADHD and their caretakers to help them maximize the clinical benefit and manage the anticipated adverse outcomes [7]. Figure 1 displays pharmacist’s major roles in treating ADHD and its associated outcomes.

### 3.1. ADHD Education

Previous research has highlighted several knowledge gaps regarding ADHD and its treatment and suggested that further education to raise awareness of ADHD is needed [29]. With sufficient knowledge provided to patients with ADHD or their guardians, they are more adherent to their medications and eventually help mitigate the symptoms of ADHD [30]. Medication adherence is critical in controlling disease, resolving temporary conditions, ensuring long-term health, and improving patients’ quality of life [31]. Ultimately, pharmacists could play a major role in educating the guardians or parents of ADHD patients, answering the physicians’ inquiries in choosing the right agent to treat the patient, and designing the pharmacotherapeutic regimen and monitoring plan [32]. ADHD education by pharmacists can help patients to better understand ADHD management, particularly in terms of medication side effects, the onset of action, the instructions on administration, monitoring frequency, and requirements [9].

Educational interventions may come in a spectrum of forms and use varied platforms [33]. In a study by Lavielle et al. (2018), educational interventions were classified into two types: those through electronic platforms and those through hard copies of resources [34]. An educational intervention that covers available treatment options for ADHD and engages patients and caretakers in composing the treatment regimens is more likely to improve the quality of ADHD care [30]. A lack of such engagement and shared decision making could eventually increase concerns regarding the prescribed treatment and the medication regimen burden [35].

A study carried out among Toronto pharmacists observed that the majority of pharmacists were not very familiar with ADHD medications and management, indicating that many pharmacists still need further support to equip them with the essential knowledge and skills [36]. To build capacity among pharmacists to undertake this essential educational role, it is critical to provide disease-specific training that includes the latest evidence-based clinical guidelines [37]. As with other healthcare professionals, pharmacists are required to take part in continuous professional development (CPD) and stay up to date on disease-specific information and recommendations that help them provide optimal patient care [38].

### 3.2. Optimization of Medications in a Collaborative Practice Model

The optimization of medicines assists patients in terms of medication adherence, long-term management, multiple morbidities, and polypharmacy [39]. Examples of the approaches used by pharmacists include (i) reviewing patients’ lists of prescribed medications, (ii) meticulously discussing every medicine with the patient, and (iii) reviewing the patient’s medications together with their clinical medical records and discussing the intended outcomes of the reviews [40].

Non-adherence to prescribed medications among ADHD patients is a multifactorial issue, and one of the reasons is that patients are not fully aware of the optimal way to manage the disease and the prescribed pharmacotherapy [41]. It is predicted that it takes approximately four months for patients to discontinue their medications in children with ADHD aged 12 to 18 years old, so targeted interventions and education need to be provided to children and caregivers to promote adherence [42]. It is the role of pharmacists to be able to provide knowledge to caretakers as well as patients about the negative consequences of a missed dose, explaining the proper method of consuming prescribed medications and what to avoid, especially the improper use of medications and potential overdose situations [43]. As for younger ADHD patients, a simpler explanation about their illness and the prescribed medications is appropriate, while as they get older, a more complex explanation of the essential aspects of monitoring and long-term medication management becomes critical to helping them cope with their daily functional demands [37].

Pharmacists can collaborate with psychiatrists to provide independent follow-up in ADHD clinics. Integrating clinical pharmacists into an ADHD specialty clinic improves patients’ adherence to ADHD treatment [44]. Initially, pharmacists visited patients collaboratively with a psychiatrist and then conducted independent follow-ups with the patients. Over the three years of intervention, there was a significant increase in the number of appointments, an improvement in adherence to monitoring blood pressure and heart rate, and more willingness to adhere to a clinic policy that required patients’ signatures for stimulant medication.

Furthermore, the experience of a pharmacist-led interprofessional ADHD clinic in a large integrated healthcare system found that the clinic has successfully shown improvements in terms of cost and efficiency of operations, and a large portion of patients reported positive outcomes in terms of fulfilling the ADHD clinic service, achieving a stabilization point, and not being re-referred to the same facility within 1 year [45]. Patients’ experiences were more likely to improve when they were involved in deciding on the best care for themselves [46]. Moreover, it is encouraged to provide patients with a simpler regimen, as it can promote better medication adherence [47,48]. Apart from that, the improvement in patients’ medication adherence also helps to lower the treatment cost for patients due to their increased reliance on relatively lower-cost ADHD medications. This is consistent with the most recent NICE guideline, which states that pharmacists should devise a more cost-effective medication regimen when optimizing medication use [39].

### 3.3. Management and Monitoring of Side Effects

Some individuals may develop unfavorable adverse effects, necessitating administration, dose, or medicine modification. Pharmacists may find the ADHD rating scale useful in closely monitoring the effects of medicine on the core symptoms of inattention, hyperactivity, and impulsivity [49]. The side effects of pharmacological medicines used to treat ADHD in children and adolescents ranged from sleeplessness and decreased appetite to headaches, tiredness, sorrow, and euphoria [50]. Adults who used methylphenidate or amphetamines typically had headaches, reduced appetite, and sleeplessness [51]. In the United Kingdom, a recent study of the prescribing trends for ADHD medications and the reporting of the incidence of adverse drug reactions (ADR) revealed that guanfacine had the greatest number of reports for serious or fatal ADR incidents. In contrast, methylphenidate recorded the fewest severe or fatal ADR events in the same year [52].

From the pharmacists’ point of view, a Canadian study highlighted that about 67% of pharmacists believe that psychostimulants are the most common cause of initial insomnia. Interestingly, community pharmacists had significantly higher knowledge of ADHD compared to hospital pharmacists, which might be caused by the lower number of ADHD patients encountered by hospital pharmacists [36]. According to the Canadian ADHD Resource Alliance (CADDRA) recommendations, atomoxetine might benefit ADHD individuals experiencing worsening sleep issues, although it could still induce early insomnia [53]. Melatonin was the non-prescription medication for sleep disorders most commonly recommended by pharmacists [36]. Accordingly, the European ADHD Guidelines Group (EAGG) recommended providing melatonin above the normally dispensed range (up to 5–6 mg per night) to help improve total sleep time [54].

A pharmacist must recognize how stimulants influence appetite and growth in children with ADHD. Children using stimulants should have their height and weight assessed regularly, such as semi-annually [45]. Both stimulants and atomoxetine cause rare but serious cardiovascular effects, such as increased blood pressure and heart rate [51]. Stimulants should be used with caution in ADHD patients with pre-existing cardiac problems [53]. It is important for pharmacists to the check blood pressure and heart rate at follow-up sessions, examine medication adherence, evaluate adverse drug effects, assess mood changes, and adjust the treatment plan, which may include ADHD, anxiety, and depression medications. Follow-up appointments were usually planned every two to four weeks until the patient had reached a stable dosage, after which they were scheduled every one to three months [55].

### 3.4. Provision of Remote and Virtual Care (Telepsychiatry Pharmacy Practice)

The use of technology in healthcare, including pharmacy practice, is well established and covers many medical specialties [56]. The feasibility of an online intervention to help parents take care of their children with ADHD has been proven [57]. It started to gain more interest during the COVID-19 lockdown because it was associated with worsening ADHD symptoms, including increased activity, irritation, and disruptive behavior [58]. As a result, guidelines were issued to help healthcare professionals, including pharmacists, provide ADHD pharmacotherapy services while adhering to strict measures during COVID-19 [54].

According to the EAGG guidelines, pharmacists are required to educate families about the importance of reporting any symptoms related to the heart, such as palpitations, shortness of breath, or chest discomfort, as soon as possible. Furthermore, the guidelines recommend that all appointments involving initial assessments of children with ADHD should continue, but they should be conducted remotely using videoconferencing or telephone, in line with the telepsychiatry guidelines [54]. Table 3 provides an overview of the recommendations made in the guidelines for the treatment of ADHD during the pandemic, arranged in accordance with the management considerations highlighted in the EAGG guidelines [54].

Telepsychiatry refers to using remote technology to assess and intervene while providing mental health services [59,60,61]. The “text message”-based intervention (three messages per week for two weeks) was found to be acceptable for the management of ADHD patients based on the participating parents’ responses [57]. In a review conducted to investigate the usefulness of telepsychiatry in ADHD therapy, the findings suggested that telepsychiatry might be a viable option for providing assessment- and evidence-based pharmacological treatment for ADHD patients [62]. In addition, a randomized control trial evaluated the feasibility of a telephone-assisted self-help intervention (TASH) for caregivers to manage ADHD patients who were prescribed methylphenidate, and the findings showed that caregivers were satisfied with the positive impact of the TASH intervention on medication adherence and outcomes [63]. It is important to note that a pharmacist can follow the best practices of videoconferencing for telemental health, which cover considerations about technology settings, physical examinations, and telepsychiatry prescribing [64].

Non-adherence to medication usually occurs in late adolescence and is associated with various adverse health outcomes, such as higher chances of injuries and emergency department visits [65]. A telepsychiatry pharmacy practice can help increase medication adherence among ADHD patients, especially those with transport problems. Currently, this service is considered a novel service for ADHD treatment [45]. A study was conducted to compare the outcomes of a pharmacist-led interprofessional ADHD clinic versus a psychiatrist-led interprofessional ADHD clinic [45]. In the study, pharmacists started and managed the patients’ medications via telephone until the patients were stable. After the patients were stable, they were transitioned back into primary care and were not added to the psychiatrist’s caseload. This innovative intervention yielded successful outcomes, with only 3.7 telephone follow-ups needed per successful patient, with about one follow-up every two weeks. In addition, this intervention was also cost-effective since the clinic saved a huge amount of money and had more time to cater to more patients.

## 4. Associated Outcomes of Pharmacists’ Involvement in ADHD Care

### 4.1. Achievement of Clinical Outcomes

In a retrospective evaluation of introducing the collaborative model of pharmacists’ involvement in providing regular counseling and psychological services as a part of an interprofessional team with psychiatrists, the findings showed improvements in adherence to clinical monitoring and compliance with the medication-taking policy [44]. This initial evaluation highlighted that pharmacists could directly contribute to ensuring medication safety and increasing awareness regarding the optimal use of ADHD medications. In addition, pharmacists’ roles can help increase support for self-management and self-awareness about the condition, initial screening and point of referral, and providing clinical consultation and feedback to ADHD patients, thus helping them to achieve therapeutic outcomes [66]. It is worth highlighting that telemental health consultations have been associated with greater symptomatic and functional improvements and distress reduction compared to children with ADHD receiving standard treatments [62]. This could be beneficial when considering the mode of engagement with ADHD patients and how it can affect the achievement of clinical outcomes.

### 4.2. Improvement of Quality of Life

ADHD has a serious and negative impact on a child or adolescent’s health-related quality of life (HRQOL). It has a moderate impact on the physical domain while having a severe impact on the psychosocial domains, which include emotional, social, and school interactions [67]. There is growing evidence to suggest that a successful and effective treatment may be able to improve a patient’s quality of life [68]. Therefore, the role of healthcare providers, including pharmacists, is critical in initiating, intensifying, and monitoring ADHD treatment to ensure effectiveness and quality of life improvement.

### 4.3. Cost-Effectiveness

It is well established that patients with ADHD tend to have higher direct medical costs compared to individuals without ADHD. According to one German analysis, the main drivers of this increased cost are inpatient care, psychiatrists and psychotherapists, and comorbidities such as anxiety, substance use disorders, and obesity [69]. In this scenario, pharmacists are suited to provide pharmaceutical care services and follow-up to empower patients to adhere to their medications, avoid medication-related harms, and manage the comorbidities that may arise [7]. Furthermore, several analyses highlighted differences in the cost-effectiveness of ADHD treatment modalities in different case scenarios [70,71]. Therefore, through their contribution to treatment decision making, pharmacists could help guide the choice of cost-effective treatment plans for patients with ADHD.

## 5. Conclusions

In collaboration with other healthcare providers, pharmacists are ultimately responsible for providing comprehensive ADHD education and support, selecting the most appropriate treatment agent, optimizing the pharmacotherapeutic regimen, and developing a monitoring plan. Pharmacists’ involvement in ADHD care is associated with better chances of achieving clinical outcomes, improving quality of life, and increasing the chances of a cost-effective treatment plan. Though they are capable of performing these responsibilities, it is imperative that they stay current on updates to the ADHD clinical standards and recommendations.

## Figures and Tables

**Table 1:** Overview of the treatment approaches for ADHD.

ADHD Treatment			
Non-Pharmacological Approaches		Pharmacological Approaches	
Behavioral Approaches	Dietary Approaches	Pharmacological Class	Examples
Psychoeducation (Parent training programs, recommended as the first-line intervention)	Elimination diet, which involves removing artificial food colorings, additives, sweeteners, and sugar. The foods listed in the few-foods diet (FFD) include two types of meat, vegetables, and fruits as well as oil and water.	Stimulants	- Methylphenidate(it is considered a first-line pharmacotherapy for children aged 5 years and older and young people with ADHD) - Amphetamine
Cognitive behavioral therapy (CBT)	Supplementation diet, which involves the addition of the following: Amino acids such as methionine; Essential fatty acids such as omega 3; Vitamins; Minerals such as iron, zinc, and magnesium.	Non-stimulants	- Atomoxetine - Guanfacine - Clonidine

**Table 2:** Essential information concerning approved ADHD pharmacotherapy.

Approved Pharmacological Options					
	Examples	Mechanism of Action	Available Formulations	Major Adverse Effects	Parameters to be Monitored
**Stimulants**	Methylphenidate	Dopamine and norepinephrine transporter inhibition, agonist activity at the serotonin type 1A receptor, and redistribution of the vesicular monoamine transporter 2	Available as chewable tablets, liquid formulations, and transdermal patches. They are provided in short-acting and long-acting formulations. Long-acting formulations are associated with better medication adherence, while short-acting formulations offer flexibility in drug dosing and titrations.	- Decreased appetite - Sleep disturbances - Increased blood pressure and pulse - Headaches and irritability - Stomach pain	- Height - Weight - Pulse - Blood pressure
	Amphetamine	Inhibition of dopamine and norepinephrine transporter, vesicular monoamine transporter 2, and monoamine oxidase activity			
**Non-stimulants**	Atomoxetine	Norepinephrine reuptake inhibition	Available as capsules and oral solution	Decreased appetite, headache, stomach pain, nausea, vomiting, sleep disturbances, and increased blood pressure and pulse	- Suicidality - Clinical worsening - Unusual changes in behavior - Pulse - Blood Pressure
	Clonidine	Agonism at alpha-2 adrenergic receptors (leading to enhanced noradrenergic neurotransmission)	Available as tablets and transdermal patch	- Somnolence/sedation - Fatigue - Hypotension - Bradycardia - Irritability - Insomnia	- Pulse - Blood pressure
	Guanfacine		Available as tablets		

**Table 3:** Recommendations for ADHD management during the pandemic, according to management considerations.

Management Considerations	Recommendations
School	During online classrooms, monitoring children with ADHD should be given a higher priority. Consider the emotional well-being of the children.
Family	Parents should receive behavioral parenting training to reduce disruptive behavior at home. Monitor children at home and seek support from accessible healthcare professionals, such as pharmacists, through online platforms. Inform prescribers if their children encounter adverse events, including cardiovascular events.
Pharmacists	Assist in initiating pharmacotherapy upon confirming an ADHD diagnosis. Recommend that caregivers refrain from altering doses above what is prescribed. Communicate with the caregivers to perform a remote clinical cardiovascular evaluation (BP and HR). Consider dispensing melatonin above the therapeutic range (5–6 mg every night) to overcome sleep disorders, if necessary.

## Data Availability

The data are available from the corresponding author upon request.

## Abbreviations

Attention deficit hyperactivity disorder	ADHD
Diagnostic and Statistical Manual of Mental Disorders, Fifth Edition	DSM-V
Cognitive behavioral therapy	CBT
Neurofeedback	NF
Adverse drug reactions	ADR
European ADHD Guidelines Group	EAGG
Canadian ADHD Resource Alliance	CADDRA
Health-related quality of life	HRQOL
